# Safety of intravenous iron isomaltoside for iron deficiency and iron deficiency anemia in pregnancy

**DOI:** 10.1007/s00404-020-05509-2

**Published:** 2020-04-08

**Authors:** Jan Wesström

**Affiliations:** 1 Department of Obstetrics and Gynecology, Center for Clinical Research Dalarna, Falun Hospital, Falun, Sweden; 2 Department of Women’s and Children’s Health, Uppsala University, Uppsala, Sweden

**Keywords:** Pregnancy, Iron deficiency, Intravenous iron, Iron isomaltoside, Safety

## Abstract

**Purpose:**

To evaluate the efficacy and safety for mother and child of using intravenous iron isomaltoside (IV-IIM) during pregnancy.

**Methods:**

Using an appointment register, we retrospectively identified all pregnant women who received a single dose of 1000 or 1500 mg IV-IIM in the maternity ward of Falu Hospital and subsequently gave birth between August 6, 2013 and July 31, 2018. Women who received IV-IIM (case group) were individually matched with pregnant women who did not receive IV-IIM (control group) by delivery date, maternal age (± 2 years), and parity. Adverse drug reactions (ADRs), demographic characteristics, hemoglobin and s-ferritin counts, pregnancy and delivery complications, and infant data (APGAR score, pH at umbilical artery, birthweight, birth length, intrauterine growth restriction and neonatal ward admission). Data were obtained from electronic patient charts. SPSS was used for descriptive statistics.

**Results:**

During the 5-year period, 213 women each received a single administration of IV-IIM. Ten (4.7%) ADRs occurred during IV-IIM administration. All ADRs were mild hypersensitivity reactions, abated spontaneously within a few minutes, and did not recur on rechallenge. No association between IIM dose and ADR frequency was noted. Maternal and fetal outcomes, including hemoglobin counts at delivery and postpartum, were similar in the case and control groups.

**Conclusion:**

These results support the convenience, safety, and efficacy of a single high-dose (up to 1500 mg) infusion of IV-IIM for iron deficiency or iron deficiency anemia during pregnancy.

## Introduction

Maternal and postpartum iron deficiency anemia (IDA) can lead to low birth weight, preterm birth, perinatal and neonatal mortality [1], lactation problems [2], postpartum depression [3, 4], and early-childhood iron deficiency (ID) [5–7]. Preventive oral iron supplementation reduces the incidence of maternal ID or anemia by 57–70% [8] but is insufficient for severe IDA [9]. After the first trimester, intravenous (IV) iron has been shown to be safe and effective for treating maternal IDA (hemoglobin [Hb] < 90 g/L) or nonanemic ID with severe symptoms [10–12].

All IV formulations may cause allergic reactions, which mostly occur within 24 h of infusion, are self-limited, resolve without treatment, and rarely recur with rechallenge [13]. Most reactions to IV iron formulations are complement activation-related pseudo allergy (CARPA) reactions to infusion nanoparticles [14]. Common symptoms of these self-limited acute hypersensitivity reactions include flushing and acute chest or back tightness, without hypotension, wheezing, stridor, or periorbital edema [15]. Life-threatening hypersensitivity reactions with IV iron preparations are very rare [16]. For IV iron isomaltoside (IV-IIM), this risk is estimated to be 0.06% [17].

An IV iron formation used in Europe, IV-IIM can be administered rapidly at high doses (e.g., maximum single dosage of 2000 mg over 30 min). Several clinical trials found that IV-IIM was well tolerated and improved markers of IDA, but few studies have described IV-IIM use in pregnancy. Therefore, the primary goal of this retrospective case–control study is to assess safety of IV-IIM in pregnant women. A secondary goal is to compare the efficacy between standard care and IV-IIM among anemic (and/or in some cases iron deficient) pregnant women after the first trimester.

## Methods

It was decided not to obtain informed consent since used retrospective data were de-identified and, therefore, considered not to be harmful for the individual patient. It was also considered to be combined with major practical problem reaching all the studied women and that informed consent obtained retrospectively could cause distress or confusion to the pregnant participants who may already be quite anxious. It could also be argued that patients might be negatively affected by the feeling that that their physician does not really know if given treatment was safe. This approach was approved by the Central Ethical Review Board of Sweden (DNR 4-2017).

Pregnant women from the beginning of the second trimester until a few days before delivery who received IV-IIM at the Maternity Ward of Falu Hospital and subsequently gave birth between August 6, 2013 and July 31, 2018 were retrospectively identified through an appointment register (*n* = 213 women). For the control group, an equal number of pregnant women who did not receive IV iron were matched to the case group by date of delivery, maternal age ± 2 years, and parity. For both case and control groups, data for the mother and infant, including the occurrence of adverse drug reactions (ADRs), demographic characteristics, occurrence of co-medication (eg. oral iron, folic acid, vitamin B12), Hb and s-ferritin counts, and complications during pregnancy and delivery, were obtained from the electronic patient charts. The Statistical Package for the Social Sciences (SPSS) was used for descriptive statistics.

For the case group, according to the study protocol, indications for IV-IIM iron were symptoms associated with anemia and/or iron deficiency, Hb < 100 g/L and/or s-ferritin < 20 µg/L. The laboratory criteria were met in most cases. No woman has had a prior IV-IIM infusion. Women who weighed < 75 kg at their first visit (typically, gestational week 10–12) to the maternity care unit received 1000 mg IV-IIM during a minimum of 15 min. Women with body weight ≥ 75 kg on the same occasion received 1500 mg IV-IIM during a minimum infusion of 30 min. IIM was diluted in 100–500 ml of saline (typically, 250 ml). Infusions were administered by three trained midwifes at the specialist Antenatal Care Department at Falu Hospital. The pregnant woman was placed in a secluded quiet room and directed to lie on her back on a comfortable bed. She was informed about the possibility of a mild, quickly reversible reaction. She was also told that the midwives would be familiar with and well prepared to handle the reaction. The midwife was in the patient’s room during the first few minutes of the infusion and remained in direct communication thereafter. The patient was equipped with an alarm button. A responsible obstetrician was present at or in direct communication with the department. Resuscitation facilities were available, and an anesthesiologist was always available on call. Seven women received iron administrations due to severe symptoms of Willis–Ekbom’s disease (WED), formerly known as restless legs syndrome (RLS). IV iron can be an alternative in treatment of WED during pregnancy since dopamine agonists is contraindicated during this period [18].

## Results

Demographics were generally similar between the case and control groups (Table 1). For education, the significant difference between groups arose from a greater number of individuals of unknown educational background among cases compared to controls (51 vs 30 patients). Significantly more women in the case group with lower levels of iron stores suffered from psychiatric conditions (*P* = 0.005). There was no statistical difference between the case and control groups in the prevalence of the other reported comorbidities (*P* = 0.59). Since the number of women with African or Middle East origin is high (82 vs 63 in case vs control group) where hereditary anemia is common, it is interesting to evaluate the prevalence of such conditions. Three women in the case group were reported to have thalassemia compared to one woman in the control group (non-significant).

**Table 1:** Demographic information for case and control groups

Characteristic	Case group (*n* = 213)	Control group (*n* = 213)	*P*
Age, average (years)	29.2	29.2	0.93
Parity, average	1.7	1.6	0.70
Ethnicity, *n*			0.10
Caucasian	124	145	
African descent	61	41	
Middle East	21	22	
Asian (except Middle East)	5	5	
Other or unknown	2	0	
Education level, *n*			0.03
Less than lower education	0	4	
Lower education^a^	23	27	
Middle education^b^	95	99	
Higher education^c^	44	53	
Unknown	51	30	
Body mass index, average (kg/m^2^)	26.0	26.1	0.90
Comorbidity^d^, *n*			0.59
Cardiovascular	7	2	
Hypothyroidism	12	8	
Diabetes	2	1	
Asthma	16	13	
Rheumatoid arthritis	7	1	
Psychiatric conditions	44	23	0.005

All average values are means

^a^Elementary school

^b^High school

^c^University education

^d^Without psychiatric conditions

Each of the 213 women in the IV-IIM group received one single infusion of IV-IIM during their second or third trimester of pregnancy. Non of the woman in the case group had a repeated IV-IIM further on in the pregnancy, 10 (4.7%) ADRs took place during the IV-IIM administration. Of the ten cases with ADR, four cases were in patients of Caucasian descent (3.2% of all Caucasians) and six cases were in patients of African, Middle Eastern, or Asian descent (7.3%) (*P* = 0.20 between ethnic groups). No serious treatment-related adverse events were observed.

Maternal and fetal outcomes were generally similar between the case and control groups (Table 2). The Hb levels were clinically and numerically nearly equal in both groups (116 vs 121 g/L, *p* 0.001) at delivery. Hb levels increased substantially after IV-IIM administration, with a satisfactory proportion of patients achieving an increase in Hb of at least 20 g/L. In the case group, the mean Hb increased from 97 g/L before treatment to 116 g/L at delivery. Among controls, the mean Hb decreased from 125 g/L at the first antenatal visit to 121 g/L at delivery. The case group had a higher number of cesarean sections, although the difference did not reach the level of statistical significance, indicating a type 2 error (Table 3).

**Table 2:** Pregnancy and delivery outcomes in case and control groups

Outcome	Case group (*n* = 213)	Control group (*n* = 213)	*P*
Hypertension, *n*	2	0	0.24
Preeclampsia/HELLP, n	5^a^	6	1
s-Ferritin at first visit antenatal clinic, average (µg/L) (L^−1^)^b^	13.1	37.6	< 0.001
Hemoglobin value, average (g/L)			
First visit to antenatal clinic	114	125	< 0.001
Before treatment	97		
Last value before delivery	116	121	< 0.001
Post-partum (1–2 days after delivery)	104	107	0.33
Gestational age at delivery, average (days)	275.4	277.3	0.12
Mode of delivery, *n*			0.25
Spontaneous vaginal	147	168	
Assisted vaginal, vacuum	9	5	
Primary cesarean	24	16	
Secondary cesarean	17	15	
Blood loss during delivery, average (mL)	553^c^	500	0.19
Birth weight, average (g)	3504	3589	0.13
Birth length, average (cm)	49.8	50.4	0.01
Apgar score at 5 min, average			1
≤ 7	9	9	
> 7	202	202	
pH at umbilical artery, average (*n* = 113)	7.26	7.26	0.76
Neonatal ward admission, *n*	12^d^	9	0.51

All average values are means

^a^Missing data for three patients

^b^s-ferritin level < 5 L^−1^ was recorded as 5 L^−1^

^c^Missing data for two patients

^d^Missing data for one patient

**Table 3:** Treatment-related adverse event and ethnicity in case group (*n *= 213) treated with iron isomaltoside

Outcome	Case group (*n* = 213)
Mild adverse event, *n* (%) (“Fishbane reaction”)	10 (4.7)
Treatment-related severe event	0 (0)
**“Fishbane reaction” and ethnicity**	*p* = 0.20
Caucasians	4 (3.2)
Africa, Middle East, Asia	6 (7.3)

## Discussion

During the 5-year observation period, 10 (4.7%) mild ADRs occurred during IV-IIM administration to 213 women. Maternal and fetal outcomes, including Hb levels, were similar in the case and control groups. The results of our study support the conclusion that a single high-dose infusion (up to 1500 mg) of IV-IIM during pregnancy is effective and safe for treating IDA in this population. As has been reported previously [19], we found no association between the dose of iron and the frequency of ADRs.

In a meta-analysis from 2018, the prevalence of treatment-related mild ADRs in pregnancy with three different intravenous iron treatments (not IIM) was variable with median prevalence from 2.2 to 6.7% [20]_._ A 2017 poster from England described 4 cases (3.3%) of mild, quickly reversible ADRs during IV-IIM administration among 121 women who received IV-IIM. In that report, there was no difference in the incidence of ADRs between patients receiving less than or more than 1000 mg of iron [21]. Similar to those previous findings, we observed that ten patients (4.7%) experienced ADRs. Nine of the ten patients with ADRs went on to complete the infusion. In one patient, the IV-IIM infusion was stopped and not restarted. This case was handled by an inexperienced doctor.

In the present report, 185 patients (86.9%) received 1000 mg of IIM, 26 patients (12.2%) received 1500 mg of IIM, and 2 patients (0.9%) received 500 mg of IIM. At the start of the study, the recommendation at our clinic was that the maximum dose of IIM should be 1000 mg. This recommendation changed during the study, and the maximum dose was increased to 1500 mg. The first larger dose was administered on February 26, 2107. From this date, the proportion of 1000 vs 1500 mg IIM was 60% and 40%, respectively.

Severe hypersensitivity reactions during iron infusions are very rare but can be life threatening and are a major medical emergency. All the infusion-related reactions in our cohort were mild and typically included face rash (flush), nausea, and sometimes anxiety. Symptoms abated spontaneously, without therapy, over a few minutes and did not recur on rechallenge. If a mild hypersensitivity reaction (HSR) occurred, the midwifes stopped the infusion for at least 10–15 min and assessed the response. Vital signs were checked, and symptoms decreased and disappeared after a few minutes. The iron infusion was restarted at no more than 50% of the initial infusion rate. After approximately 5–10 min, the infusion rate was increased again.

There seems to be an unjustified fear among healthcare professionals of giving even modern IV iron preparations to pregnant women. Adverse reactions are mistakenly described as anaphylaxis, prompting unnecessary interventions and misleading clinicians about the toxicity profile of IV iron. The confidence and competence provided by regular training should help reduce any anxiety on the part of the healthcare professional and, conceivably, the risk of HSR. In fact, the anxiety of the healthcare professionals giving IV drugs in itself has been shown to increase the risk of HSR [22].

By law, each county council in Sweden must have at least one drug committees with representatives having pharmaceutical and medical expertise. These representatives give recommendations based on scientific evidence and experience. All county councils and regions issue local regulations on pharmaceutical activities and working methods. The drug committee in Dalarna County has concluded that iron preparations should only be given in an environment where resuscitation facilities are available, so that in the rare occasion, a patient develops an allergic reaction, he or she can be treated immediately. Therefore, according to this committee, any unit capable of managing acute reactions to vaccines can administer IV iron. Accordingly, IV iron can easily be given to outpatients at primary care facilities.

Psychiatric comorbidities were significantly more common among pregnant women with iron deficiency anemia. Previous observational epidemiological studies have reported findings regarding the association between anemia and the risk of maternal depression in both antepartum and postpartum depression. Several plausible biological mechanisms have been posited to explain the link. It is clear that brain iron status influences emotional behaviors and mental health by altering neurotransmitters and their receptors (eg. dopamine, norepinephrine, serotonin, GABA, cytochrome C) [23]_._ Our results support the association.

One weakness in the study is that many women (17%) in the case group received their iron infusion after gestational week 36. Thus, the time interval between iron administration and the last Hb measure was too short to enable a substantial Hb rise. Another possible factor that could affect the Hb and ferritin levels at baseline is that 7 women in the study had Willis–Ekbom’s Disease. IV iron is used in this population when their s-ferritin levels are below 50 µ/L, regardless of their Hb value. We decided to keep this group of women in the study, because the primary goal of the study was to evaluate safety among pregnant women receiving IV-IIM. Our data, and those of others, support the call for large prospective studies of IV iron for the treatment of maternal IDA.
